# The m6A methyltransferase METTL3 controls epithelial-mesenchymal transition, migration and invasion of breast cancer through the MALAT1/miR-26b/HMGA2 axis

**DOI:** 10.1186/s12935-021-02113-5

**Published:** 2021-08-21

**Authors:** Chengpeng Zhao, Xiaoling Ling, Yunxia Xia, Bingxue Yan, Quanlin Guan

**Affiliations:** 1 Department of Medical Oncology, The First Hospital of Lanzhou University, 730000 Lanzhou, People’s Republic of China; 2 The First School of Clinical Medicine, The First Hospital of Lanzhou University, 730000 Lanzhou, People’s Republic of China; 3 Department of Oncology Surgery, The First Hospital of Lanzhou University, No. 1, Western Donggang Road, Chengguan District, Gansu 730000 Lanzhou, People’s Republic of China

**Keywords:** Breast cancer, METTL3, MALAT1, MiR-26b, Epithelial-mesenchymal transition

## Abstract

**Background:**

Previous studies have revealed the key functions of N6-methyladenosine (m6A) modification in breast cancer (BC). MALAT1 as a highly m6A modified lncRNA associated with cancer development and metastasis, but the functional relevance of m6A methyltransferase and MALAT1 in BC is still unknown. Here, our study investigated the effects of the novel m6A methyltransferase METTL3 on epithelial-mesenchymal transition (EMT) in BC via the MALAT1/miR-26b/HMGA2 axis.

**Methods:**

Firstly, we collected clinical BC samples and cultured BC cells, and detected mRNA and protein levels in the human samples and human cell lines by RT-qPCR and Western blot, respectively. Then, the binding of MALAT1 and miR-26b and the targeting relationship between miR-26b and HMGA2 were examined by dual-luciferase assay. Moreover, the binding of MALAT1 and miR-26b was tested by RNA pull down and RNA immunoprecipitation (RIP) assays. Methylated-RNA immunoprecipitation (Me-RIP) was used to detect the m6A modification level of MALAT1. The interaction of METTL3 and MALAT1 was detected by photoactivatable ribonucleoside-crosslinking immunoprecipitation (PAR-CLIP). Finally, effects on invasion and migration were detected by Transwell.

**Results:**

In BC, the level of miR-26b was consistently low, while the levels of METTL3, MALAT1 and HMGA2 were high. Further experiments showed that METTL3 up-regulated MALAT1 expression by modulating the m6A modification of MALAT1, and that MALAT1 could promote the expression of HMGA2 by sponging miR-26b. In BC cells, we found that silencing METTL3 could inhibit EMT and tumor cell invasion by suppressing MALAT1. Furthermore, MALAT1 mediated miR-26b to target HMGA2 and promote EMT, migration, and invasion. In summary, METTL3 promoted tumorigenesis of BC via the MALAT1/miR-26b/HMGA2 axis.

**Conclusions:**

Silencing METTL3 down-regulate MALAT1 and HMGA2 by sponging miR-26b, and finally inhibit EMT, migration and invasion in BC, providing a theoretical basis for clinical treatment of BC.

**Supplementary Information:**

The online version contains supplementary material available at 10.1186/s12935-021-02113-5.

## Introduction

Breast cancer (BC) is the most frequent cancer occurring in women worldwide, with approximately 268,600 estimated new cases diagnosed among US women in 2019, with 41,760 women dying from the disease. There are various types of BC, including Luminal A BC, Luminal B BC, Triple-negative/basal-like BC, HER2-enriched BC and Normal-like BC [1]. Based on the molecular classification, various therapeutic interventions are available for BC [2]. Chemotherapy plays an important role in BC management, where tamoxifen is frequently used either alone or in combination with other chemotherapeutic agents [3]. Although improvements in surgical techniques, chemotherapy, and radiation delivery, along with supportive care, have improved the survival and quality of life for patients with BC, regional and distant recurrence remain common [4]. A range of other therapeutic drugs have been developed to promote a better clinical outcome of BC patients [5–7], but new targeted therapies are desperately needed.

Epithelial-mesenchymal transition (EMT) is a cell-biological program defined by the loss of epithelial characteristics and the acquisition of a mesenchymal phenotype [8]. As a broadened definition rather than a stereotypical program, EMT is believed to be associated with many developmental, wound healing, fibrosis, and cancer processes [9]. Excessive epithelial cell proliferation, invasion and transition aroused by EMT are hallmarks of carcinomas [10].

Long non-coding RNAs (lncRNAs) are transcripts exceeding 200 nucleotides, but with no protein-coding capacity [11]. Recent attention focused on the effects of modifications on non-coding RNA have shown that m6A modification of RNA is critically important [12]. Metastasis-associated lung adenocarcinoma transcript-1 (MALAT1) is a evolutionarily conserved 7–9 kbp lncRNA, which is known to be associated with cancer development and metastasis. MALAT1 includes several m6A sites modified in a high percentage of transcripts from different BC cell lines. What’s more, its overexpression has been identified as an important factor in BC development [13]. Studies have shown that the expression level of METTL3 was up-regulated both in BC the human samples and human cell lines [14]. Jin et al. found that METTL3 up-regulates MALAT1 at the level of transcription through provoking a higher level of m6A modification, and showed that the METTL3/YTHDF3 complex could improve the stability of MALAT1 [15]. microRNAs (miRNAs) mediate the expression of many target genes and play important roles in many cancers including BC. Previous findings showed that miR-203 was highly upregulated in BC tissues and in ER-positive BC cell lines, and that miR-203 promotes BC cell proliferation in vitro and is required for tumor growth in a xenograft model [16]. Further, evidence has shown that exosomal MALAT1 derived from human hepatic cells regulates miRNA-26 A: MALAT1 acts as an miRNA sponges and reduced the expression of miR-26b in hepatic stellate cells [17]. Since miR-26b normally acts as a tumor suppressor, its down-regulation is often associated with development of carcinomas and poor prognosis [18]. Especially, in triple negative breast cancer (TNBC), down-regulated miR-26b reduced cell growth and colony formation [19].

Based on our human samples results, the level of miR-26b was consistently low, while the levels of METTL3, MALAT1 and HMGA2 were high in BC. Though no former publication presents evidence showing any relationship between miR-26b-5p, MALAT1 and HMGA2, our in vivo and in vitro experiments showed that METTL3 promotes expression of MALAT1 by modulating m6A modification of MALAT1 and MALAT1 could sponge absorb miR-26b and suppress miR-26b expression in BC cells. And further we used starBase for analysis, which predicted that miR-26b-5p has a binding site on HMGA2 3’ UTR and that miR-26b-5p should specifically inhibit HMGA2 by targeting it. To investigate the mechanism of EMT regulation in BC, especially the role of METTL3 as a m6A methyltransferease in the development of BC through its effects on the METTL3/MALAT1/miR-26b axis, we sought in this study to identify the downstream target genes of miR-26b and their influences on EMT in BC.

## Materials and methods

### Bioinformatic analysis

Starbase website (http://starbase.sysu.edu.cn/index.php) and RNA22 website (https://cm.jefferson.edu/rna22/) were used to predict the targeting sites for the interaction between MALAT1 and miR-26b.

### Collection of clinical samples

From October 2015 to October 2017, 68 cases of primary BC were operated in the First Hospital of Lanzhou University, and the BC tissue samples and the corresponding adjacent tissue samples were collected. The BC patients ranged in age from 27 to 69 years old, with a median age of 51 years. All participants received routine treatment but without chemotherapy before operation, and all patients were confirmed by pathological diagnosis after the operation. This study was approved by Ethics Committee of the First Hospital of Lanzhou University, and carried out following the Declaration of Helsinki. The pathological diagnosis of patients is shown in Additional file 1: Table S1.

### Cell culture and grouping

The BC cancer cells, MCF-7(ZQ0071), MDA-MB-231(ZQ0118), MDA-MB-468(ZQ0373) and normal mammary epithelial cells MCF-10 A were provided by the Shanghai Zhongqiao Xinzhou Co., Ltd. (Shanghai, China) and ATCC company, respectively. Cells were cultured in Dulbecco’s Modified Eagle’s medium (DMEM) with 10 % FBS, in a constant temperature incubator at 37 ℃ and 5 % CO_2_. After adhering to the culture dishes, the dells were digested with 0.25 % trypsin (Hyclone Company, Logan, Utah, USA) and passaged, and the logarithmic growth period cells were used for subsequent experiments. All cell lines have been tested for mycoplasma after their purchase and STR analysis was performed every month.

According to the needs of the experiment, the cells were divided into the following groups: sh-NC (transfected with gene interference negative control plasmids), sh-MALAT1 (transfected with gene interference MALAT1 interference plasmids), sh-NC + oe-NC (transfected with gene interference negative control plasmids and overexpression negative control plasmids), sh-METTL3 + oe-NC (transfected with interference METTL3 plasmid and over expression negative control plasmid), sh-NC + oe-MALAT1 (transfected with negative control plasmids and overexpression MALAT1 plasmids), sh-METTL3 + oe-MALAT1 (transfected with interference METTL3 plasmid and overexpression MALAT1 plasmid), NC-mimic (transfected with miR-26b overexpression negative control plasmid), miR-26b mimic (transfected with miR-26b overexpression plasmid), NC-inhibitor (transfected with miR-26b interference negative control plasmid), miR-26b inhibitor (transfected with miR-26b interference plasmid), sh-NC + NC-inhibitor (transfected with negative control plasmids of transfected gene interference and miR-26b interference plasmid), sh-MALAT1 + NC-inhibitor (transfected with interference MALAT1 plasmid and miR-26b interference negative control plasmid), sh-MALAT1 + miR-26b inhibitor (transfected with interference MALAT1 plasmid and miR-26b interference plasmid), NC-mimic + oe-NC (transfected with miR-26b negative overexpression control plasmid and negative overexpression control plasmid), miR-26b mimic + oe-NC (transfected with miR-26b overexpression plasmid and negative overexpression control plasmid), miR-26b mimic + oe-HMGA2 (transfected with miR-26b overexpression plasmid and HMGA2 overexpression plasmid), and sh-METTL3 + oe-HMGA2 (transfected with interference METTL3 plasmid and overexpression HMGA2 plasmid). All interference and overexpression plasmids were designed and provided by Invitrogen (Calsbad, CA, USA). The lipofectamine 2000 kit (product No. 11,668,019, Thermo Fisher Scientific Inc., CA, USA) was used for cell transfection. 1 × 10^5^ cells were inoculated into each well in a six-well plate and cultured until cell confluence reached 60–70 %. Then, 250 µL of serum-free Opti-MEM medium (Gibco, Grand Island, New York, USA) was used to dilute 4 µg of target plasmids together with 10 µL of Lipofectamine 2000, and then mixed with light agitation. Next, the mixture was added to the culture system. After 6 h in culture, the medium was replaced with complete culture medium. After a further48 hours, cells were collected to detect the transfection efficiency for subsequent experiments [20, 21].

### Quantitative real-time PCR (qRT-PCR)

Total RNA was extracted from tissues or cells with TRIzol (Invitrogen) method following the manufacturer’s protocol. RNA purity was examined by Nanodrop2000 micro-UV spectrophotometer (1011U, Nanodrop, USA). RNA was reversely transcribed into cDNA following protocols of the TaqMan MicroRNA Assays Reverse Transcription primer (4,427,975, Applied Biosystems, USA) and PrimeScript RT reagent kit (RR047A, Takara, Japan). All primers were designed and synthesized by TaKaRa (Additional file 2: Table S2). According to a reaction system prepared by the fast SYBR Green PCR kit (Applied Biosystems), quantitative PCR was detected by an ABI7500 quantitative PCR instrument (7500, ABI, USA). GAPDH and U6 were used as internal reference genes of MALAT1 and miR-26b, respectively. The expression levels of target genes were calculated by the relative quantitative method (2^−ΔΔCT^ method). ΔΔCt = ΔCt experimental group − ΔCt control group, where ΔC_t_ = Ct _(target gene)_−Ct _(internal reference)_, and the relative transcription level of target gene mRNA = 2^−ΔΔCt^. Each sample was set with three replicates and each test was repeated three times.

### Western blot (WB)

Total protein in tissue or cell was extracted by RIPA lysate solution containing PMSF (P0013C, Beyotime, Shanghai, China). The prepared cells were incubated on ice for 30 min, and then the supernatant was centrifuged (4 ℃, 8000 g) for 10 min. A BCA test kit was used to detect the total protein concentration (P0012, Beyotime). Fifty µg protein samples were dissolved in 2 × SDS sample buffer and boiled for 10 min, and SDS-PAGE gel electrophoresis was performed followed by transfer of proteins to a PVDF membrane. After blocking in 5 % non-fat milk for 1 h, PVDF membranes were incubated overnight with primary antibodies: METTL3 (1:1000, ab19552, Abcam, Cambridge, UK), HMGA2 (1:1000, ab207301, Abcam), E-cadherin (1:500, ab15148, Abcam), Snail (1:500, ab82846, Abcam), Vimentin (1:2000, ab137321, Abcam), N-cadherin (1:1000, ab18203, Abcam), and GAPDH (ab19485, 1:2500, Abcam). Then, PVDF membranes were incubated for 1 h with corresponding HRP labeled goat-anti-rabbit IgG H&L (HRP) (ab97051, 1:2000, Abcam). According to the instructions of the ECL detection kit (product No. BB-3501, Amersham, UK), equal amounts of liquid A and B were mixed in the dark and then dripped onto the PVDF membranes. The film was photographed using a specific image analysis system (Bio-Rad company, USA), and analyzed using Quantity-One (v4.6.2) software. The relative protein content was expressed by the gray value of corresponding protein bands/the gray value of internal reference bands. The mean of triplicate experiment results was calculated [20, 21].

### Immunohistochemistry

The tumor tissue samples were fixed with 10 % neutral formalin solution, dehydrated, embedded in paraffin, and sectioned by an ultrathin sectioning machine. The sections were then dewaxed with xylene, rehydrated with graded alcohol, and incubated with 3 % hydrogen peroxide to block the activity of endogenous peroxidase. The sections were boiled in 10 mM sodium citrate (pH 6.0) for 30 min, sealed in 10 % normal goat serum for 15 min, and then incubated overnight with antibodies against METTL3 (1:500, ab195352, Abcam), HMGA2 (1:500, ab207301, Abcam), Snail (1:2000, ab224731, Abcam), N-cadherin (1:1000, ab18203, Abcam), and Vimentin (1:500, ab137321, Abcam) in a wet chamber at 4 ℃. The next day, the sections were washed with PBS and incubated with secondary antibody for 1 h at room temperature prior to visualization as above.

### Transwell

The Matrigel (YB356234, Shanghai Yu Bo Biotech Co., Ltd., Shanghai, China) preserved at − 80 ℃ was melted at 4℃ overnight. Then, 200 µL serum-free medium and 200 µL Matrigel were fully mixed at 4 ℃ to dilute the Matrix gel. Next, 50 µL diluted matrix gel was added to each Transwell plate upper chamber, placed in the incubator, and incubated for 2–3 h for solidification. Next, 2 × 10^4^ MCF-7 cells were added to the upper chamber of each well, and 800 µL medium containing 20 % FBS to the lower chamber, and incubated at 37 ℃ for 24 h. Next, the Transwell plate was immersed in 10 % formaldehyde for 10 min and then rinsed three times with clear water. The fixed cells were stained with 0.1 % crystal violet (Solarbio Co., Beijing, China) for 30 min, and the cells on the surface were then wiped off with cotton ball. Finally, the samples were observed, photographed, and counted under a microscope. In the Transwell migration experiment, there was no need to deposit the matrix glue, and the incubation time was 16 h. Cells were counted in four randomly selected fields of view [22].

### Methylated-RNA immunoprecipitation (Me-RIP)

Total RNA was isolated from BC cells by the Trizol method. The mRNA in total RNA was separated and purified by PolyATtract® mRNA Isolation Systems (product No. A-Z5300, Eide Technology Co., Ltd., Beijing, China). An IP buffer (20 mm Tris, pH 7.5,140 mM NaCl, 1 % NP-40, 2 mM EDTA), along with anti-m6A antibody (1:500, ab151230, Abcam) or anti-IgG antibody IgG (ab109489, 1:100, Abcam) were added and incubated with protein A/G magnetic beads for 1 h for binding. The purified mRNA and bead-antibody complex was added to the IP buffer along with ribonuclease inhibitor and protease inhibitor and incubated overnight at 4 ℃. The RNA was eluted with elution buffer and purified by phenol chloroform extraction. MALAT1 expression level was assessed by qRT-PCR, and each experiment was repeated three times [23, 24].

### Photoactivatable ribonucleoside enhanced crosslinking immunoprecipitation (PAR-CLIP)

BC cells were incubated with 200 mM 4-thiopyridine (4SU) (Sigma-Aldrich, St. Louis, MO, USA) for 14 h, and then cross-linked with 0.4 J/cm^2^ at 365 nm. The lysates were immunoprecipitated with METTL 3 antibody and incubated overnight at 4 ℃. RNA was labeled with [g-^32^P]-ATP and observed by autoradiography. The precipitate was digested by protease K to remove the protein, and the relative quantitative expression of MALAT1 was examined using qRT-PCR assay.

### Dual luciferase reporter assay

To construct luciferase reporter vector, HMGA2-3′UTR and MALAT1 cDNA fragments containing miR-26b binding site were inserted into pGL3 plasmids. We constructed the binding site mutations of the HMGA2-3′UTR-MUT and MALAT1-MUT fragments by the point mutation method and inserted them into pGL3 plasmid. All the vector sequences were confirmed by gene sequencing. Then, pGL3-MALAT1, pGL3-MALAT1-MUT, pGL3-HMGA2-3′UTR, and pGL3-HMGA2-3′UTR-MUT recombinant vectors and Renilla internal reference plasmids were transfected into HEK-293 cells by liposome transfection with miR-26b mimic or NC-mimic, respectively. Cells were collected and lysed after 48 h of transfection. A luciferase detection kit (K801-200, Biovision, Milpitas, CA, USA) was used for detection of reporter genes by a Dual-Luciferase gene analysis system (Promega, Madison, WI, USA). Renilla luciferase was regarded as the reference gene, and the relative luciferase (RLU) activity of target reporter genes was calculated as the ratio of RLU activity of firefly luciferase to that of renilla luciferase [25].

### RNA-pull down

50 nM biotin labeled bio-miR-26b-probe and bio-NC probe (Jinkairui Bioengineering Co., Ltd., Wuhan) were used to transfect the cells, which were collected after 48 in culture. The cells were placed on ice in Pierce IP lysis buffer (Thermo Fisher Scientific Inc.) for 30 min, and then centrifuged to collect the supernatant cell lysate. Each lysate was added with an equal amount of beads (Thermo Fisher Scientific Inc.) and incubated overnight at 4 ℃. The next day, the precipitates were collected by centrifugation, washed twice with precooled cracking buffer, and then eluted with high salt buffer. Finally, the supernatant was collected by centrifugation. The RNA combined with miR-26b was purified by the Trizol method and the enrichment of MALAT1 was detected by qRT-PCR [25].

### RNA Immunoprecipitation (RIP)

Cells were lysed in an equal volume RIPA lysate (p0013B, Beyotime) for 30 min. Lysates were centrifuged at 4 ℃, and 14,000 rpm for 10 min to obtain the supernatant. The binding of MALAT1 to Ago2 was detected by a RIP kit (Millipore, USA). In brief, the coprecipitation system was added with 50 µL magnetic beads, and then resuspended in 100 µL RIP wash buffer, followed by addition of 5 µg rabbit anti-Ago2 antibody (ab186733, 1:50, Abcam). The reaction system was incubated at 4 ℃ for at least 6 h. After washing in buffer, the bead-antibody complex was resuspended with 900 µL RIP wash buffer and 100 µL cell supernatant. The samples were placed on the magnetic base to collect the bead-protein complex, digested by protease K, and then the RNA was extracted for subsequent PCR detection. Rabbit anti-IgG (ab172730, 1:100, Abcam) was used as negative control, and the experiment was repeated three times.

### Tumorigenesis assay in nude mice

Eighteen BALB/C female nude mice (SLAC Laboratory Animal Co., Ltd., Shanghai, China) aged 4–5 weeks and weighing 15–18 g were randomly assigned into three groups of six mice. The MCF-7 cell lines stably transfected with sh-NC + oe-NC, sh-METTL3 + oe-NC and sh-METTL3 + oe-HMGA2 were selected for subcutaneous establishment of the BC cell line MCF-7 as xenografts in the nude mice. For this purpose, MCF-7 cell lines in the logarithmic growth stage were prepared into a suspension with a concentration of about 1 × 10^7^ cells/ml. The prepared cell suspension was injected into the left armpit of the mice, and the subsequent tumor growth was recorded. After four weeks, mice were killed by cervical dislocation. Tumor tissues were resected and weighed with a balance. The expression of proteins in tumor tissues was examined using Western blot.

For construction of the model of lung metastasis of BC, 3 × 10^6^ MCF-7 cells infected with sh-NC + oe-NC, sh-METTL3 + oe-NC or sh-METTL3 + oe-HMGA2 were injected into nude mice via a tail vein. After 5 weeks, the pathological changes of lung tissue were observed by hematoxylin and eosin staining (HE staining) and the metastasis of tumor nodules were observed.

All the above animal experiments had been approved by the hospital animal protection and use committee, and conformed to management and use principles.

### HE staining

Lung tissue of nude mice was collected, fixed in 10 % neutral formalin, then embedded in paraffin and dewaxed with xylene. Then, the tumor sections were stained with hematoxylin, washed with distilled water, immersed in 95 % ethanol, stained with eosin, hydrated with gradient ethanol, dehydrated with xylene, dried and fixed with neutral resin, and then observed under optical microscope.

### Statistical analysis

All data were represented as the mean ± standard deviation. All analyses were completed with SPSS 24.0 software (IBM, Chicago, IL, USA). The comparisons between paired or unpaired groups were analyzed with paired or unpaired t-test, respectively. The data of multiple groups were compared with Tukey’s test-corrected one-way analysis of variance (ANOVA). Data among groups at different times were compared by Bonferroni test-corrected two-way ANOVA or repeated measurement ANOVA. *P* < 0.05 was statistically significant.

## Results

### METTL3 upregulates MALAT1 by modulating m6A modification of MALAT1

**Fig. 1:**
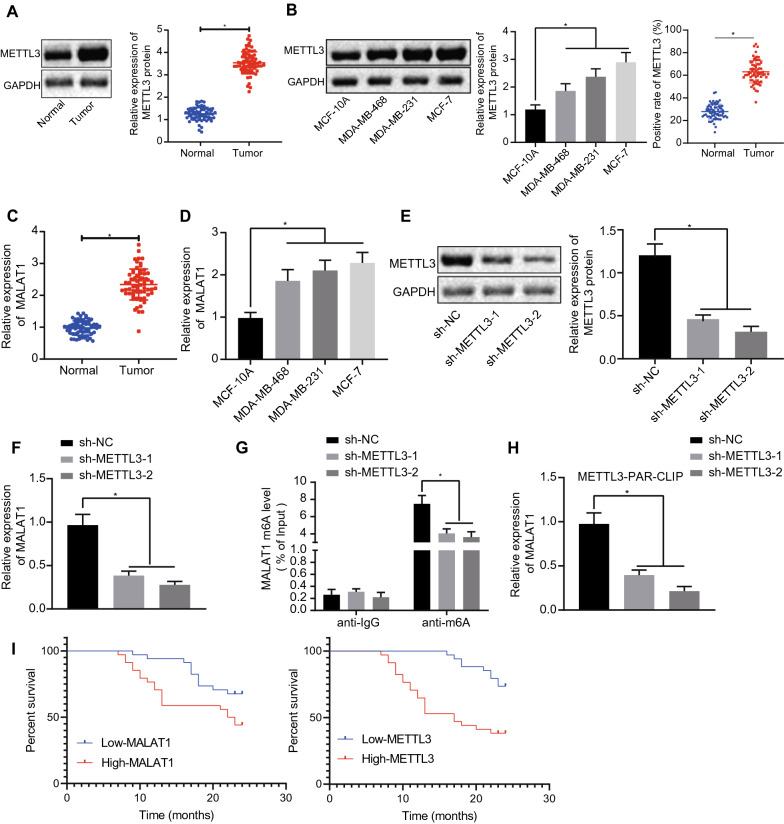
METTL3 promotes expression of MALAT1 by modulating the m6A modification of MALAT1 in BC cells. **A** The METTL3 expression in BC tissues detected by Western blot analysis (n = 68). **B** The expression of METTL3 in BC cells was detected by Western blot analysis and in BC tissues detected by immunohistochemistry. **C** The MALAT1 expression in BC tissues measured by qRT-PCR (n = 68). **D** The expression of MALAT1 in BC cells was detected by qRT-PCR. **E** Western blot detection results of METTL3 expression level after METTL3 silencing. **F** qRT-PCR results of METTL3 expression after METTL3 silencing. **G** After silencing METTL3, m6A modification level of MALAT1 was detected in MCF-7 cells by Me-RIP. **H** The combination of METTL3 and MALAT1 detected by PAR-CLIP. I: Kaplan-Meier survival curves of BC patients (n = 68). *Significant difference (*P* < 0.05). The above data were expressed as mean ± standard deviation. Paired t-test was used to compare between BC and adjacent tissues samples, and unpaired t-test was used between the other two groups. One-way ANOVA with Tukey’s post-test was used for data comparison among multiple groups. Kaplan-Meier survival method was used to calculate the survival rate. All experiments were repeated three times

Published studies have revealed that methyltransferase METTL3 can increase MALAT1 expression by promoting the m6A modification of MALAT1 [15]. Besides, METTL3 can promote the development and progression development of BC [14], and MALAT1 is highly expressed in malignant BC [26, 27]. To evaluate the possibility that methyltransferase METTL3 is involved in the regulation of the m6A modification of MALAT1 in BC, we first detected the expression of METTL3 and MALAT1 in BC. Our results showed that the expression levels of METTL3 and MALAT1 were significantly up-regulated in BC (Fig. 1 A–D). In order to study effect of METTL3 on the expression level of MALAT1, we silenced METTL3 in MCF-7 cells. The expression of METTL3 declined significantly after METTL3 was silenced, indicating that our silencing procedure was successful (Fig. 1E, F). These results suggest that METTL3 may be involved in the regulation of MALAT1 expression.

By measuring the m6A modification level of MALAT1 in BC cells with Me-RIP, we observed that m6A modification of MALAT1 was significantly down-regulated after METTL3 was silenced (Fig. 1G). The PAR-CLIP test detected the combination of METTL3 and MALAT1, and results (Fig. 1H) showed that after METTL3 was silenced, the interaction of METTL3 and MALAT1 was significantly reduced. Additionally, Kaplan-Meier survival analysis of clinical data showed that expressions of METTL3 and MALAT1 were inversely correlated with BC patients’ survival, and survival analysis of clinical data showed that the high expression of METTL3 and MALAT1 were always evident in patients with poor survival (Fig. 1I).

### Silencing of METTL3 can inhibit EMT, migration and invasion in BC cells by restraining MALAT1 expression

**Fig. 2:**
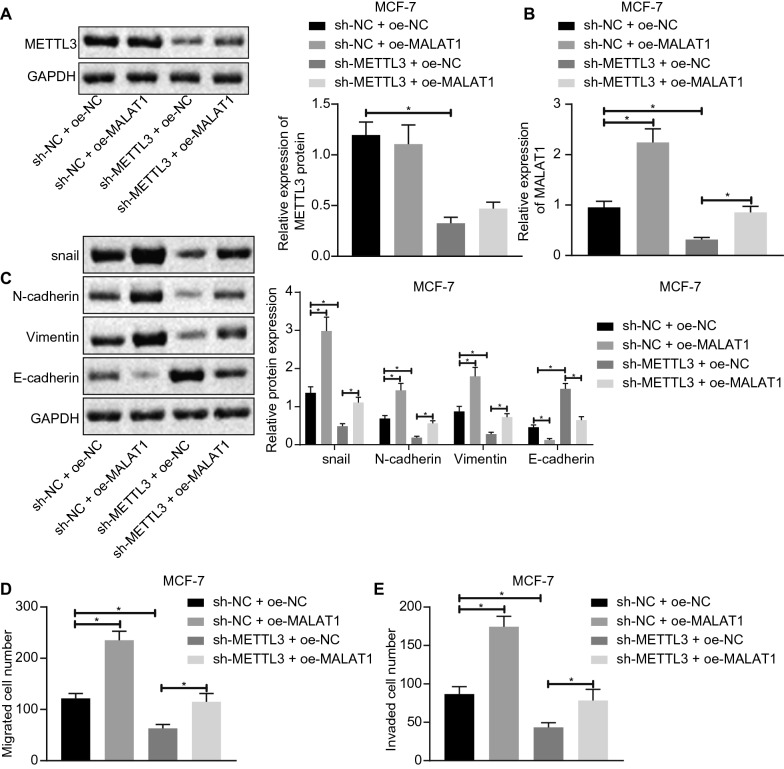
Silencing of METTL3 can inhibit EMT, migration and invasion in BC by repressing MALAT1 expression. **A** The expression of METTL3 protein was detected by Western blot analysis. **B** The MALAT1 expression was detected by qRT-PCR. **C** The EMT-related proteins expression levels in cells were detected by Western blot analysis **D** The migration in cells examined by Transwell assay. **E** The invasion in cells examined by Transwell assay. *Significant difference (*P* < 0.05). The above data were all measurement data and expressed as mean ± standard deviation. One-way ANOVA with Tukey’s post-test was used for data comparison among multiple groups. All experiments were repeated three times

To observe the effects of METTL3 on the functions of BC cells by controlling MALAT1 expression, we chose MCF-7 and MDA-MB-231 cells for further investigations. The results showed no significant difference in METTL3 expression between the sh-NC + oe-NC and sh-NC + oe-MALAT1 groups. However, compared with the sh-NC + oe-NC group, the MALAT expression was significantly increased in the sh-NC + oe-MALAT1 group, while expression of METTL3 and MALAT1 in the sh-METTL3 + oe-NC group decreased. Compared with the sh-METTL3 + oe-NC group, there was no significant difference in METTL3 expression in the sh-METTL3 + oe-MALAT1 group, while the MALAT1 expression was significantly up-regulated (Fig. 2A, B, Additional file 3: Fig. 1A, B). Further, results also showed that, compared with sh-NC + oe-NC group, the expression on BC cells of proteins related to EMT (Snail, N-cadherin, and Vimentin) were significantly higher in the sh-NC + oe-MALAT1 group, while the expression of E-cadherin protein was significantly lower. The protein expressions of Snail, N-cadherin, and Vimentin in sh-METTL3 + oe-NC group was significantly lower, while the expression level of E-cadherin was elevated. Compared with the sh-METTL3 + oe-NC group, the expression of Snail, N-cadherin, and Vimentin in sh-METTL3 + oe-MALAT1 group were significantly up-regulated, while E-cadherin was down-regulated (Fig. 2C and Additional file 3: Fig. 1C). Moreover, the results from Transwell assays showed that the migration and invasion of BC cells in the sh-NC + oe-MALAT1 and sh-METTL3 + oe-MALAT1 group was significantly enhanced, but was reduced in the sh-METTL3 + oe-NC group compared with their controls (Fig. 2D and Additional file 3: Fig. S1D, E).

### MALAT1 can sponge and inhibit miR-26b expression

**Fig. 3:**
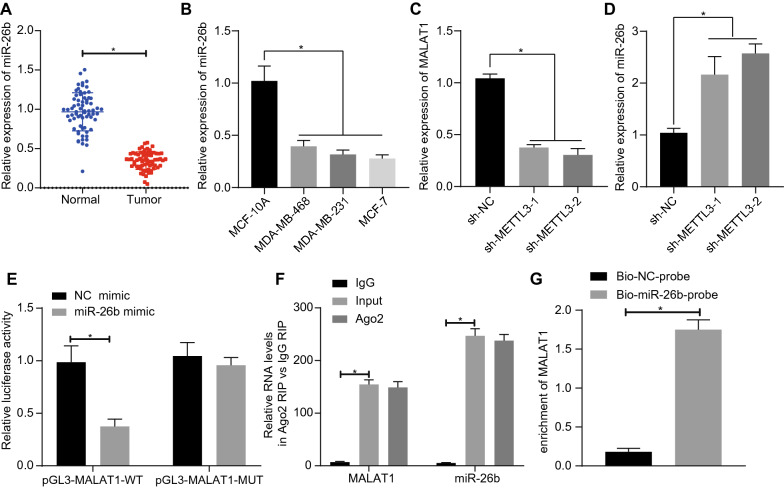
MALAT1 can sponge miR-26b and restrict miR-26b expression in BC cells.** A** The miR-26b expression in BC tissue was examined by qRT-PCR, n = 68. **B** The miR-26b expression in BC cells was detected by qRT-PCR. **C** The expression of MALAT1 detected by qRT-PCR after MALAT1 was silenced in MCF-7 cells. **D** The expression of miR-26b in MCF-7 cells was detected by qRT-PCR after knockdown of MALAT1 expression. **E** Binding of MALAT1 to miR-26b was detected by luciferase reporter gene assay. **F** The interaction of MALAT1 and miR-26b was verified by RIP assay, and the precipitated MALAT1 and miR-26b were detected by qRT-PCR. **G** The combination of MALAT1 and miR-26b was confirmed by RNA-pull down assay, and MALAT1 was detected by qRT-PCR. *Significant difference (*P* < 0.05). The above data were all measurement data and expressed as mean ± standard deviation. Paired t-test was used between BC and adjacent groups. One-way ANOVA with Tukey’s post-test was used for data comparison among multiple groups. All experiments were repeated three times

Previous studies have shown that MALAT1 can sponge miR-26b in hepatocytes and inhibit the expression of miR-26b [17]. Besides, miR-26b expression is down-regulated in BC [19]. Therefore, to estimate the regulation of MALAT1 on miR-26b in BC, we first measured the miR-26b expression in BC. Notably, miR-26b had low expression state in BC (Fig. 3A, B). Next, we silenced MALAT1 in MCF-7 cells. Intriguingly, the MALAT1 expression decreased significantly after silencing MALAT1, while expression of miR-26b increased significantly (Fig. 3C, D). We next tested whether MALAT1 could sponge miR-26b by the luciferase reporter gene assay. The luciferase signal of pGL3-MALAT1-MUT mutant was not significantly altered, while that of pGL3-MALAT1-Wt cells was significantly reduced by miR-26b mimic compared with NC-mimic (Fig. 3E). Moreover, our RIP results revealed that MALAT1 and miR-26b were pulled down by Ago2 (*P* < 0.05) (Fig. 3F). The results of RNA-pull down experiments (Fig. 3G) showed that MALAT1 was enriched in the sample pulled down by the miR-26b probe compared with the NC probe.

### MALAT1 can promote expression of HMGA2 by sponging miR-26b

**Fig. 4:**
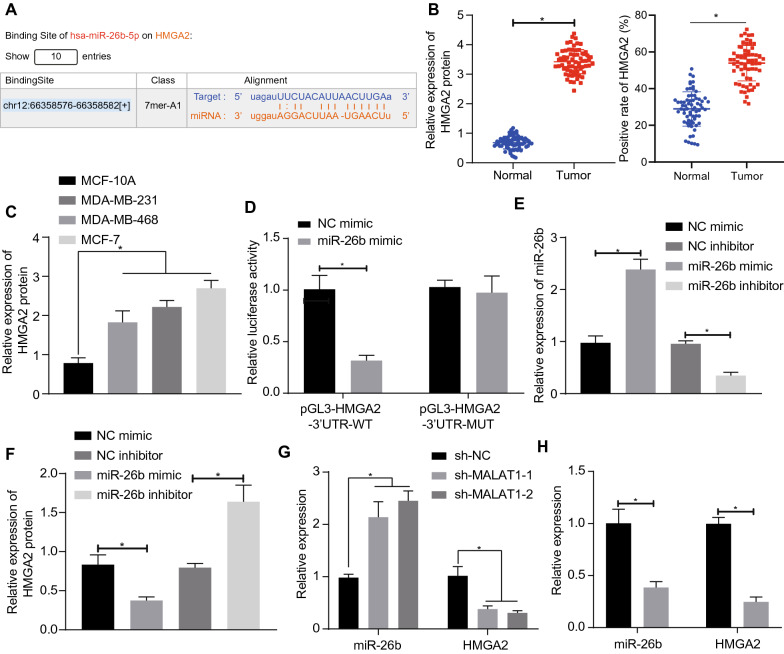
MALAT1 can promote the expression of HMGA2 by sponging miR-26b in BC cells. **A** The results from starBase database indicated binding sites between miR-26b and target HMGA2 mRNA 3’UTR. **B** The expression of HMGA2 in BC tissues was detected by Western blot analysis and immunohistochemistry, n = 68. **C** The expression of HMGA2 in BC cells was detected by Western blot analysis. **D** The binding of miR-26b and HMGA2 mRNA was confirmed by the luciferase reporter gene test. **E** The expression of miR-26b was detected by qRT-PCR. **F** The expression of HMGA2 in MCF-7 cells was detected by Western blot analysis. **G** The expression of miR-26b and HMGA2 in BC cells was detected by qRT-PCR after MALAT1 was silenced. **H** The expression of MALAT1 and HMGA2 in MCF-7 cells was detected by qRT-PCR when miR-26b was overexpressed. *Significant difference (*P* < 0.05). The above data were all measurement data and expressed as mean ± standard deviation. Paired *t*-test was used between BC and adjacent tissue samples, and unpaired t-test was used between other two groups. One-way ANOVA with Tukey’s post-test was used for data comparison among multiple groups. All experiments were repeated three times

Furthermore, the Starbase database predicted that there might be binding sites in the miR-26b and 3’UTR of HMGA2 mRNA (Fig. 4A). Consequently, we detected HMGA2 expression in BC by Western blot and immunohistochemistry. Interestingly, the HMGA2 expression was significantly elevated in BC (Fig. 4B, C). The results of luciferase reporter gene detection (Fig. 4D) showed that, compared with NC-mimic group, the luciferase signal of miR-26b mimic and pGL3-HMGA2-3′UTR co-transfection groups was significantly decreased, while that of pGL3-HMGA2-3′UTR-MUT mutant group was not significantly different. These results have proved that miR-26b is among the targets of HMGA2.

Next, to further investigate the regulation of miR-26b on HMGA2, we overexpressed and silenced miR-26b in the MCF-7 BC cell line, and detected the transfection efficiency of miR-26b by qRT-PCR. Our results (Fig. 4E) showed that the expression of miR-26b was significantly higher in the miR-26b mimic group and lower in the miR-26b inhibitor group compared with the NC-mimic and NC-inhibitor groups. The results of Western blot analysis (Fig. 4F) showed that, compared with the NC-mimic group, expression of HMGA2 was significantly lower in the miR-26b mimic group, and was significantly higher in the miR-26b inhibitor group compared with the NC-inhibitor group. These results proved that miR-26b targeted HMGA2 and inhibited HMGA2 expression in BC.

According to the above results, MALAT1 may decrease miR-26b expression by sponging and then promote expression of HMGA2. In a test of this possibility, the addition results (Fig. 4G, H) showed that miR-26b expression was significantly increased and HMGA2 expression was significantly decreased after MALAT1 was silenced.

### MALAT1 mediates miR-26b to promote EMT, migration and invasion of BC cells by controlling expression of HMGA2

**Fig. 5:**
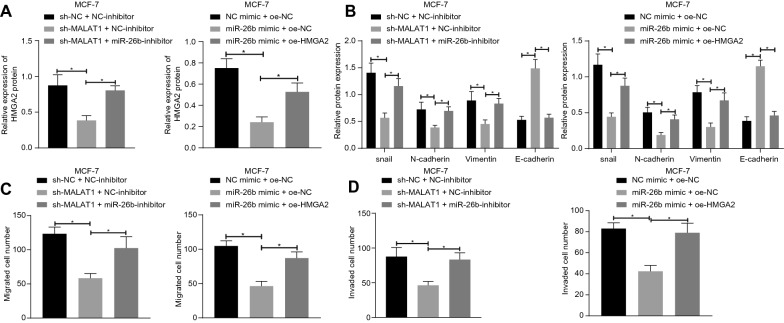
MALAT1 can promote EMT, migration, and invasion of MCF-7 cells by targeting HMGA2 with miR-26b.** A** The HMGA2 protein was detected by Western blot analysis. **B** The expression level of proteins related to EMT was detected by Western blot analysis. **C** The cell migration ability of each group was detected by Transwell assay. **D** The cell invasion ability of each group was detected by Transwell assay. *Significant difference (*P* < 0.05). The above data were all measurement data and expressed as mean ± standard deviation. Data comparison among multiple groups was analyzed by Tukey’s post-test one-way ANOVA. All experiments were repeated three times

Moreover, we further explored the roles of MALAT1 in functions of MCF-7 and MDA-MB-231 BC cells by targeting HMGA2/miR-26b. Results showed that HMGA2 expression in the sh-MALAT1 + NC-inhibitor group was significantly lower than in the sh-NC + NC-inhibitor group (Fig. 5A and Additional file 4: Fig. S2A). Besides, the expression of HMGA2 in the sh-MALAT1 + miR-26b inhibitor group was significantly higher than that of sh-MALAT1 + NC-inhibitor group. Compared with the NC-mimic + oe-NC group, the expression of HMGA2 was significantly lower in the miR-26b mimic + oe-NC group decreased, but was increased in the miR-26b mimic + oe-HMGA2 group compared with the miR-26b mimic + oe-NC group.

Other results (Fig. 5B and Additional file 4: Fig. S2B) showed that the expression of Snail, N-cadherin, and Vimentin decreased, while expression of E-cadherin increased in the sh-MALAT1 + NC-inhibitor group compared with the sh-NC + NC-inhibitor group. Compared with the sh-MALAT1 + NC-inhibitor group, Snail, N-cadherin, and Vimentin expression in the sh-MALAT1 + miR-26b inhibitor group were upregulated significantly, while E-cadherin decreased significantly. Compared with the NC-mimic + oe-NC group, the expressions of Snail, N-cadherin, and Vimentin in the miR-26b mimic + oe-NC group were significantly inhibited, while E-cadherin expression was significantly reduced. Compared with the miR-26b mimic + oe-NC group, the Snail, N-cadherin, and Vimentin expressions in the miR-26b mimic + oe-HMGA2 group were up-regulated, while the expression of E-cadherin was significantly down-regulated. Transwell assay results showed (Fig. 5C, D and Additional file 4: Fig. 2C, D) that cell migration and invasion in the sh-MALAT1 + NC-inhibitor group were significantly reduced compared with the sh-NC + NC-inhibitor group, but were increased in the sh-MALAT1 + miR-26b inhibitor group compared with the sh-MALAT1 + NC-inhibitor group. Besides, compared with the NC-mimic + oe-NC group, the migration and invasion in the miR-26b mimic + oe-NC group decreased significantly. The migration and invasion ability in the miR-26b mimic + oe-HMGA2 group were enhanced significantly compared with the miR-26b mimic + oe-NC group.

### METTL3 increases tumorigenesis and metastasis in BC modeled mice by controlling the expression of HMGA2

**Fig. 6:**
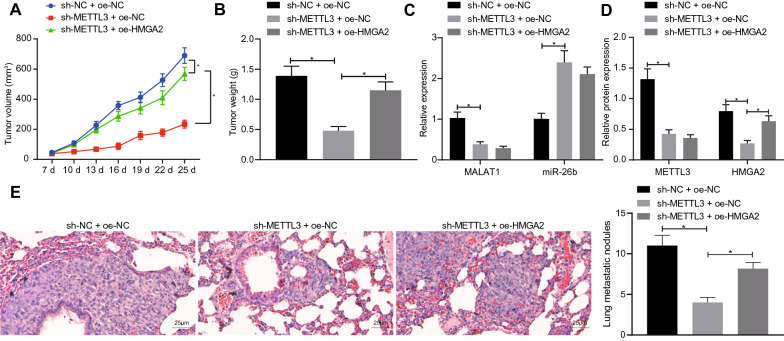
METTL3 increases tumorigenesis in BC cells by controlling HMGA2.** A** The growth curve of volume of transplanted tumor in each group. **B** The weight of transplanted tumor in each group. **C** The MALAT1 and miR-26b expression detected by qRT-PCR. **D** The expression of METTL3 and HMGA2 was detected by Western blot analysis. **E** The tumor nodules in the lung tissue of nude mice detected by HE staining (scale bar = 0.5 mm). *Significant difference (*P* < 0.05). The above data were all measurement data, expressed as mean ± standard deviation. One-way ANOVA with Tukey’s post-test was applied to data comparison among multiple groups. Repeated measurement analysis with Bonferroni post-test was used to analyze the data of each group at different time points, n = 6

Finally, we conducted tumorigenesis experiments in a mouse BC model to determine the effect of METTL3 on the growth of BC xenografts in vivo by controlling HMGA2. We found that tumor volume and weight were significantly less in the sh-METTL3 + oe-NC group than in of sh-NC + oe-NC group. Compared with the sh-METTL3 + oe-NC group, tumor volume and weight in the sh-METTL3 + oe-HMGA2 group were significantly increased (Fig. 6A, B). Besides, compared with the sh-NC + oe-NC group, the expression of METTL3, MALAT1 and HMGA2 in the sh-METTL3 + oe-NC group were lower, while miR-26b expression was significantly higher. However, the expression of METTL3, MALAT1 and miR-26b in the sh-METTL3 + oe-HMGA2 group were unaffected, although HMGA2 expression was significantly increased compared with the sh-METTL3 + oe-NC group (Fig. 6C, D). Furthermore, mouse model of lung metastasis of BC showed that down-regulation of METTL3 reduced the lung metastasis of BC, but overexpression of HMGA2 reversed this inhibition. Meanwhile, immunohistochemistry revealed that the proteins levels of Snail, N-cadherin and Vimentin decrease significantly after METTL3 knock-down, but were increased by further overexpression of HMGA2 in METTL3 knock-down cells (Additional file 5: Fig. S3). In addition, we constructed a nude mouse model of BC lung metastasis. HE staining was used to observe the tumor nodules transferred to nude mice lung tissues. The results showed that the number of tumor nodules in the sh-METTL3 + oe-NC group decreased significantly compared with sh-NC + oe-NC group. Compared with the sh-METTL3 + oe-NC group, the number of tumor nodules in sh-METTL3 + oe-HMGA2 group was significantly increased (Fig. 6E).

## Discussion

**Fig. 7:**
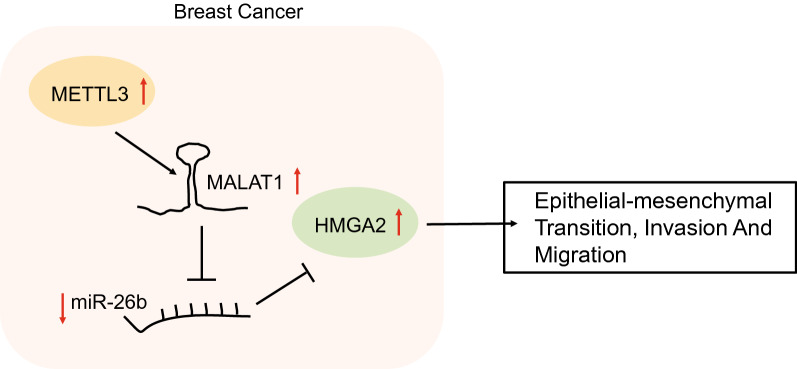
Silencing METTL3 can reduce the m6A modification of MALAT1, down-regulate the level of MALAT1, decrease the expression of HMGA2 by sponging miR-26b, and finally inhibit EMT in BC

Though is established that MALAT1 is among the most highly expressed lncRNAs in ER/PR positive type BC [28], the connection between METTL3 and MALAT1 has been unknown. To establish this relationship, we first collected 68 cases of BC tissue specimens and examined the expression levels of METTL3 and MALAT1. Results showed conspicuous overexpression both of METTL3 and MALAT1 at the levels of transcription and translation in BC tissues. To further study the influence of METTL3 expression on MALAT1, we created a METTL3 knock-down MCF-7 cell model, and confirmed that the expression and m6A modification levels of MALAT1 were both significantly down-regulated (Fig. 7).

What’s more, knowing that MALAT1 could promote EMT in BC [29], we speculated about the downstream effects of METTL3 regulation. To resolve this, we confirmed that the expression level of MALAT1 was down-regulated in conjunction with the loss of METTL3 expression in MCF-7 and MDA-MB-231 cells. It turned out that the expression level of Snail, N-cadherin and Vimentin were remarkably lower, while expression level of E-cadherin was higher in these cells. Similarly, Snail was previously shown to repress the expression of E-cadherin, while inducing the expression of mesenchymal markers such as Vimentin and N-cadherin [30]. To verify if MALAT1 promotes cell migration and invasion as described in previous work, we also conducted functional assays, which showed a promotion of invasive and metastatic characteristics [31]. Combined with the evidence found at the transcriptional level, we conclude that METTL3 knockout can reduce the EMT and invasion of BC cells through inhibiting the expression of MALAT1.

Interestingly, given that exosomal MALAT1 derived from human hepatic cells can regulate and reduce the expression of miR-26b, we supposed that the same regulation mechanism may also exist in BC [17]. To confirm this prediction, we silenced MALAT1 in MCF-7 cells and tested the miR-26b level, and also undertook RIP assay and RNA-protein pull-down experiments [32]. The results of these assays verified that MALAT1 does indeed interact with miR-26b and inhibits its expression. Using an online tool, we predicted that miR-26b has a binding site with HMGA2 mRNA 3’UTR, as likewise show by published predictions that hsa-miR-26b is an miRNA regulator of HMGA2 [33]. Also, Young et. al. found that chromosomal translocations in HMGA2 may contribute to tumorigenesis by removing the HMGA2’s 3’ untranslated region (UTR) [34]. We first designed a HMGA2 mutant and then confirmed the miR-26b target binding to HMGA2. We further overexpressed and silenced miR-26b in MCF-7 BC cells and examined the efficiency of transfection to evaluate the expected effect. The results indicated that miR-26b not only targets HMGA2, but also inhibits its expression. Since MALAT1 is known to sponge miR-26b to promote invasion and metastasis of colorectal cancer [35, 36], we expected that MALAT1 might also sponge miR-26b in BC, and that miR-26b targets and down-regulates HMGA2. Our analysis of BC cells confirmed that miR-26b was up-regulated and HMGA2 was down-regulated whenMALAT1 was silenced, while MALAT1 and HMGA2 were down-regulated when miR-26b was overexpressed. HMGA2 is a transcriptional modulator that is known to promote stemness, invasion and tumorigenicity in glioblastoma (GBM) [37]. Other evidence shows that high expression of HMGA2 is alone sufficient for tumorigenesis [34]. To better investigate the relationship between HMGA2, MALAT1 and miR-26b, we determined the effect of the MALAT1 regulatory mechanism in BC tumorigenicity. Our results demonstrated that METTL3 can promote tumorigenicity in BC by regulating HMGA2.

However, we still do not have a clear understanding of how METTL3 controls the expression of MALAT1 in BC. METTL3 mediated modification of m6A is a prevalent modification of mRNAs that regulate gene expression by modulating RNA processing, localization, translation, and eventual decay, all of which can be modulated by “writers,” “erasers,” and “readers” of this epigenetic marker [38]. Previous results have shown that the stability of MALAT1 was increased by the METTL3/YTHDF3 complex [15]. Thus, there are grounds of conducting further investigations to identify the specific effectors of m6A modification involved in regulating the level of MALAT1 expression.

## Conclusions

We can conclude that the m6A methyltransferase METTL3 controls epithelial-mesenchymal transition of breast cancer through the MALAT1/miR-26b/HMGA2 axis. our research isthe first investigation of the METTL3/MALAT1/miR-26b/HMGA2 axis in BC. Results not only clarified the regulatory pathway, but also demonstrated its downstream effect on tumorigenicity and EMT. More importantly, our results give a better understanding of the roles of METTL3 and m6A in BC, and may present a range of new therapeutic targets involving METTL3, MALAT1, miR-26b and HMGA2, especially for the treatment for triple-negative cancer.

## Supplementary Information

**Additional file 1.** The pathological diagnosis of patients.

**Additional file 2: Table S2.** Primer sequences of qRT-PCR.

**Additional file 3: Figure S1.** METTL3 silencing can inhibit EMT, migration, and invasion in MDA-MB-231 cells by restricting the MALAT1 expression. **A** The METTL3 protein detected by Western blot analysis. **B** The MALAT1 expression was measured by qRT-PCR. **C** The EMT-related proteins in MDA-MB-231 cells were detected by Western blot analysis. **D** The migration of MDA-MB-231 cells detected by Transwell assay. **E** The invasion of MDA-MB-231 cells was examined by Transwell assay. *Significant difference (*P* < 0.05). The above data were all measurement data and expressed as mean ± standard deviation. One-way ANOVA with Tukey's post-test was used for data comparison among multiple groups.

**Additional file 4: Figure S2.** MALAT1 mediates miR-26b to promote EMT, migration, and invasion in MDA-MB-231 cells by targeting HMGA2. **A** The expression of HMGA2 was detected by W Western blot analysis. **B** The expression level of EMT-related proteins in each group was examined by Western blot analysis. **C** The migration of MDA-MB-231 cells detected by Transwell assay. **D** The invasion ability of MDA-MB-231 cells was detected by Transwell assay. *Significant difference (*P* < 0.05). The above data were all measurement data and expressed as mean ± standard deviation. Data comparison among groups at different time points using repeated measurement ANOVA with Bonferroni's post-test. All experiments were repeated three times.

**Additional file 5: Figure S3.** MALAT1 mediates miR-26b to promote lung metastasis of BC cells by targeting HMGA2. Immunohistochemistry was used to detect the expression of relevant proteins in tumor tissues of nude mice. *Significant difference (*P* < 0.05).

## Data Availability

The datasets generated/analyzed during the current study are available.

## Abbreviations

BC: Breast cancer
EMT: Epithelial-mesenchymal transition
RIP: RNA immunoprecipitation
Me-RIP: Methylated-RNA immunoprecipitation
PAR-CLIP: Ribonucleoside-crosslinking immunoprecipitation
EMT: Epithelial-mesenchymal transition
MALAT1: Metastasis-associated lung adenocarcinoma transcript-1
TNBC: Triple negative breast cancer
DMEM: Dulbecco’s Modified Eagle’s medium
qRT-PCR: Quantitative Real-time PCR
Me-RIP: Methylated-RNA immunoprecipitation
PAR-CLIP: Photoactivatable Ribonucleoside enhanced crosslinking immunoprecipitation
