# Supplementary material for: The m6A methyltransferase METTL3 controls epithelial-mesenchymal transition, migration and invasion of breast cancer through the MALAT1/miR-26b/HMGA2 axis
Source: Cancer Cell Int. 2021 Aug 21;21:441. doi: 10.1186/s12935-021-02113-5 (PMC8380348; doi:10.1186/s12935-021-02113-5)
Supplement: Supplementary file 2 — Additional file 2: Table S2. Primer sequences of qRT-PCR. [file 12935_2021_2113_MOESM2_ESM.docx]

**Additional file 2: Table S2** **Primer sequences of qRT-PCR**

| Genes | Primer sequence (5'-3') |
| --- | --- |
| MALAT1 | F: 5'-CATTCGCTTAGTTGGTCTAC-3'  R: 5'-TTCTACCGTTTTTCAGCTTC-3' |
| miR-26b | F: 5'-CCGGGACCCAGTTCAAGTAA-3'  R: 5'-CCCCGAGCCAAGTAATGGA G-3' |
| GAPDH | F: 5'-CTCCTCCTGTTCGACAGTCAGC-3'  R: 5'-CCCAATACGACCAAATCCGTT-3' |
| U6 | F: 5'-CTCGCTTCGGCAGCACA-3'  R: 5'-AACGCTTCACGAATTTGCGT-3' |
