# Supplementary figures and images for: The m6A methyltransferase METTL3 controls epithelial-mesenchymal transition, migration and invasion of breast cancer through the MALAT1/miR-26b/HMGA2 axis
Source: Cancer Cell Int. 2021 Aug 21;21:441. doi: 10.1186/s12935-021-02113-5 (PMC8380348; doi:10.1186/s12935-021-02113-5)

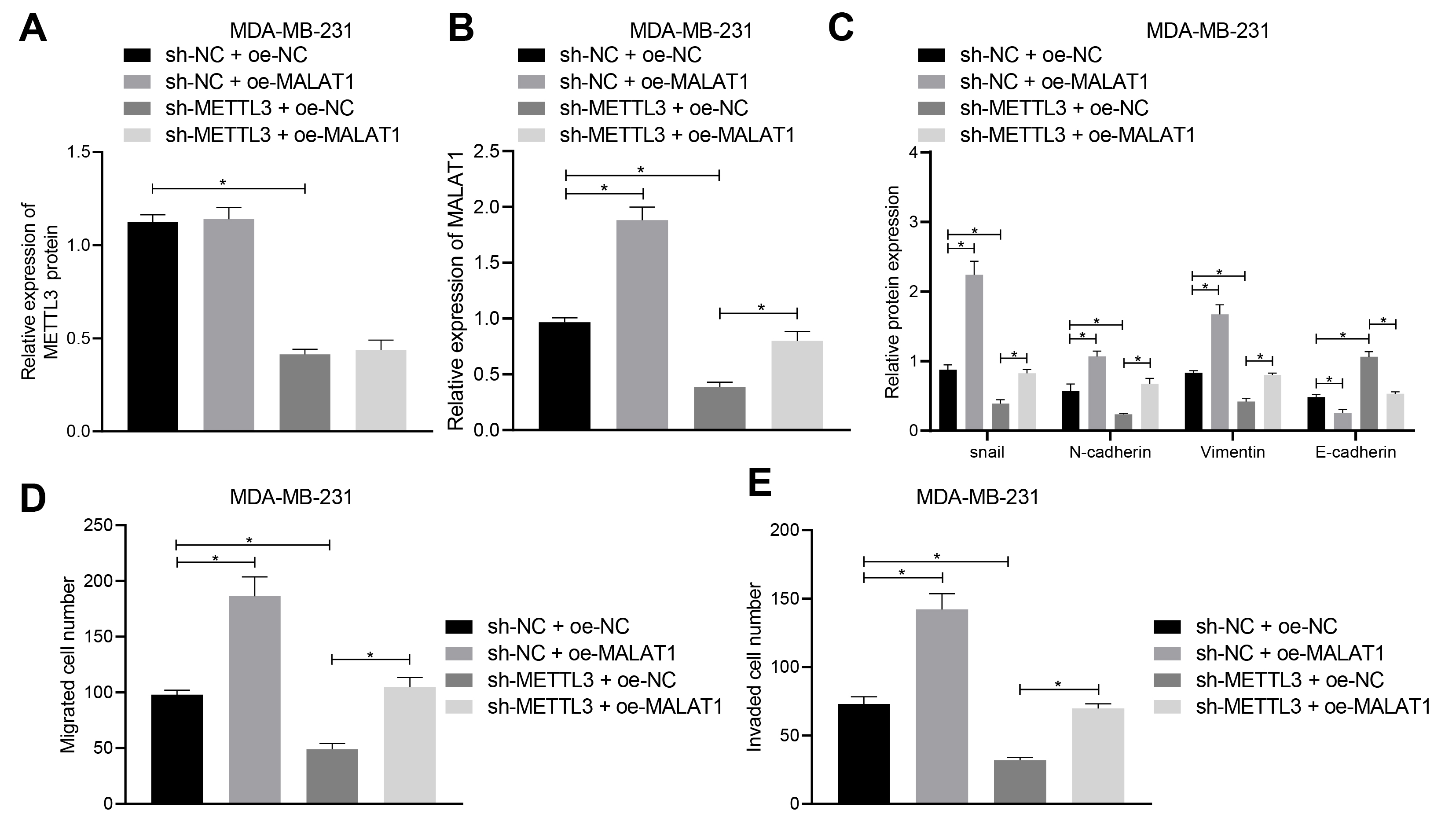

Supplement: Supplementary file 3 — Additional file 3: Figure S1. METTL3 silencing can inhibit EMT, migration, and invasion in MDA-MB-231 cells by restricting the MALAT1 expression. A The METTL3 protein detected by Western blot analysis. B The MALAT1 expression was measured by qRT-PCR. C The EMT-related proteins in MDA-MB-231 cells were detected by Western blot analysis. D The migration of MDA-MB-231 cells detected by Transwell assay. E The invasion of MDA-MB-231 cells was examined by Transwell assay. *Significant difference (P < 0.05). The above data were all measurement data and expressed as mean ± standard deviation. One-way ANOVA with Tukey's post-test was used for data comparison among multiple groups. [file 12935_2021_2113_MOESM3_ESM.jpg]

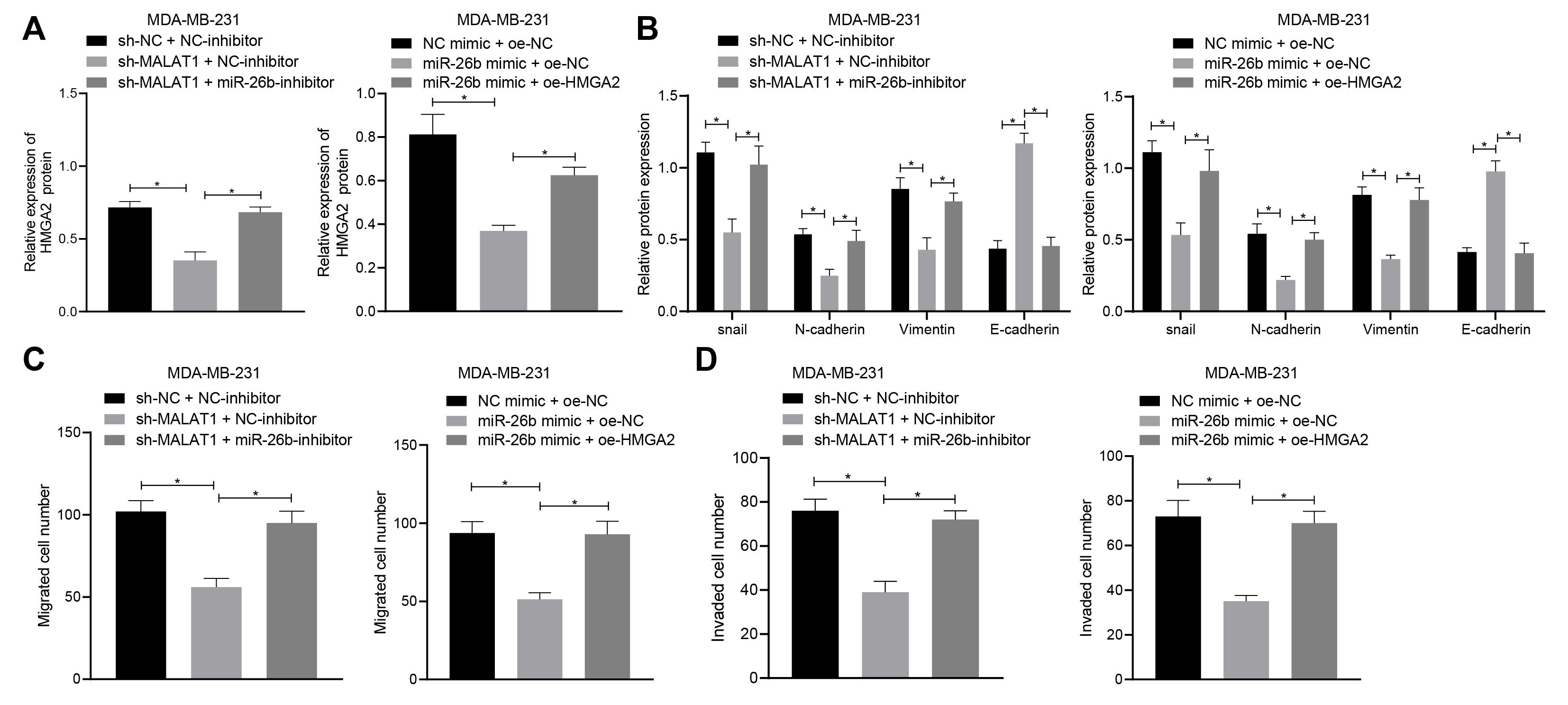

Supplement: Supplementary file 4 — Additional file 4: Figure S2. MALAT1 mediates miR-26b to promote EMT, migration, and invasion in MDA-MB-231 cells by targeting HMGA2. A The expression of HMGA2 was detected by W Western blot analysis. B The expression level of EMT-related proteins in each group was examined by Western blot analysis. C The migration of MDA-MB-231 cells detected by Transwell assay. D The invasion ability of MDA-MB-231 cells was detected by Transwell assay. *Significant difference (P < 0.05). The above data were all measurement data and expressed as mean ± standard deviation. Data comparison among groups at different time points using repeated measurement ANOVA with Bonferroni's post-test. All experiments were repeated three times. [file 12935_2021_2113_MOESM4_ESM.jpg]

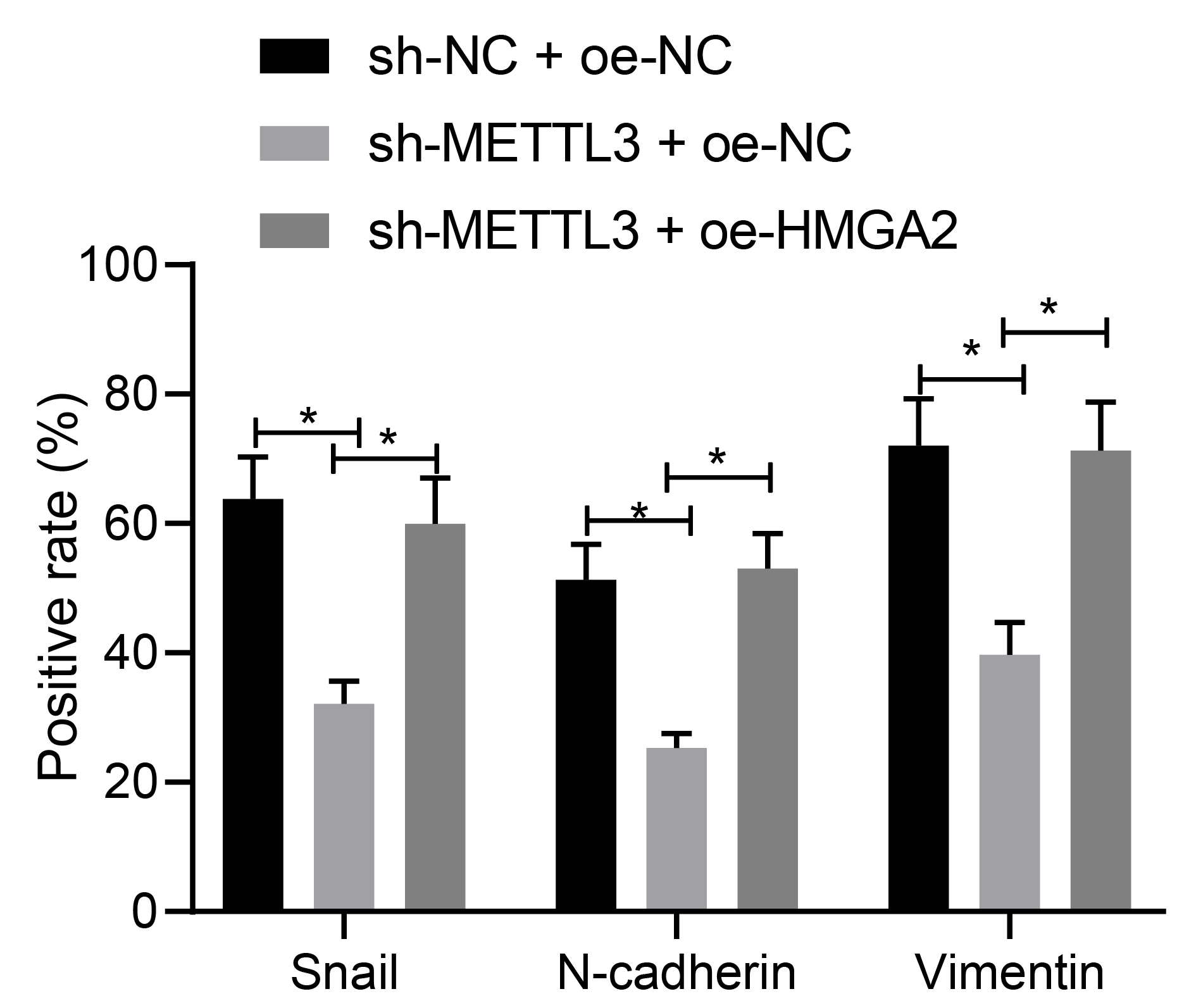

Supplement: Supplementary file 5 — Additional file 5: Figure S3. MALAT1 mediates miR-26b to promote lung metastasis of BC cells by targeting HMGA2. Immunohistochemistry was used to detect the expression of relevant proteins in tumor tissues of nude mice. *Significant difference (P < 0.05). [file 12935_2021_2113_MOESM5_ESM.jpg]
